# Intracellular *Theileria annulata* Promote Invasive Cell Motility through Kinase Regulation of the Host Actin Cytoskeleton

**DOI:** 10.1371/journal.ppat.1004003

**Published:** 2014-03-13

**Authors:** Min Ma, Martin Baumgartner

**Affiliations:** 1 Neuro-Oncology, Experimental Infectious Diseases and Cancer Research, University Children's Hospital Zürich, Zürich, Switzerland; 2 Molecular Pathobiology, University of Bern, Bern, Switzerland; 3 Graduate School for Cellular and Biomedical Sciences, University of Bern, Bern, Switzerland; University of Virginia Health System, United States of America

## Abstract

The intracellular, protozoan *Theileria* species parasites are the only eukaryotes known to transform another eukaryotic cell. One consequence of this parasite-dependent transformation is the acquisition of motile and invasive properties of parasitized cells *in vitro* and their metastatic dissemination in the animal, which causes East Coast Fever (*T. parva*) or Tropical Theileriosis (*T. annulata*). These motile and invasive properties of infected host cells are enabled by parasite-dependent, poorly understood F-actin dynamics that control host cell membrane protrusions. Herein, we dissected functional and structural alterations that cause acquired motility and invasiveness of *T. annulata*-infected cells, to understand the molecular basis driving cell dissemination in Tropical Theileriosis. We found that chronic induction of TNFα by the parasite contributes to motility and invasiveness of parasitized host cells. We show that TNFα does so by specifically targeting expression and function of the host proto-oncogenic ser/thr kinase MAP4K4. Blocking either TNFα secretion or MAP4K4 expression dampens the formation of polar, F-actin-rich invasion structures and impairs cell motility in 3D. We identified the F-actin binding ERM family proteins as MAP4K4 downstream effectors in this process because TNFα-induced ERM activation and cell invasiveness are sensitive to MAP4K4 depletion. MAP4K4 expression in infected cells is induced by TNFα-JNK signalling and maintained by the inhibition of translational repression, whereby both effects are parasite dependent. Thus, parasite-induced TNFα promotes invasive motility of infected cells through the activation of MAP4K4, an evolutionary conserved kinase that controls cytoskeleton dynamics and cell motility. Hence, MAP4K4 couples inflammatory signaling to morphodynamic processes and cell motility, a process exploited by the intracellular *Theileria* parasite to increase its host cell's dissemination capabilities.

## Author Summary

The protozoan parasite *Theileria annulata* causes the often fatal leukoproliferative disorder Tropical Theileriosis in their ruminant host animals, which is the result of widespread dissemination and proliferation of cytokine secreting, parasite-infected cells. This host cell behavior is induced by and dependent on the intracellular presence of the parasite and is reminiscent of metastatic dissemination of human cancer cells. We investigated how the intracellular parasite modulates cell motility and invasiveness, to better understand the pathogenesis of Tropical Theileriosis and to reveal conserved mechanisms of eukaryotic cell motility regulation. We found that the parasite drives host cell motility and invasiveness through the induction and activation of the host cell protein MAP4K4. We show that MAP4K4 induction is driven by the inflammatory cytokine TNFα and causes dynamic changes in the cytoskeleton of the host cell that facilitate cell motility. Thus, our findings reveal how the intracellular *Theileria* parasite can influence morphology and behavior of its host cell in a way that suits its propagation and highlight a novel function of chronic TNFα production for the pathogenesis of Tropical Theileriosis. Furthermore, our study revealed a novel aspect of inflammatory cytokine action, namely cell mobilization through the induction of the evolutionary conserved protein kinase MAP4K4.

## Introduction

*Theileria annulata* is an apicomplexan, intracellular parasite that predominately infects macrophages *in vivo*. It causes the severe leukoproliferative disorder Tropical Theileriosis in ruminants in northern Africa, the Middle East and Asia where its *Hyalomma* tick vector is endemic. It is closely related to *T. parva*, which is transmitted by the tick *Rhipicephalus appendiculatus* and predominately infects T cells to cause East Coast Fever. Hallmark of infections with *T. annulata* or *T. parva* is a host cell transformation process that results in immortalization and permanent proliferation of the infected cell population and - through paracrine stimulation – also of non-infected leukocytes [1].

*Theileria*-infected cells can be studied *in vitro* and used as a reversible model of oncogenic transformation because the parasite can be specifically eliminated by parasitocidic treatment with the drug Buparvaquone 720c (BW720c); hence transformation-dependent alterations can be determined and pathways that promote these alterations identified [2]–[4]. A series of *in vitro* and *in vivo* studies showed that *Theileria* triggers host cell motile and invasive behavior to facilitate parasite dispersion in the host animal, which is reminiscent of metastatic tumor cell dissemination [5]–[8]. TGFß was recently found to trigger a parasite-dependent invasive motility program in the host cell through the activation of Rho kinase ROCK [3], analogous to TGFß-dependent invasive migration of cancer cells [9]. Earlier studies showed that *T. annulata* infected mononuclear host cells disseminate as cytokine secreting cells throughout the body *in vivo* [6]. Cytokines expressed include the pro-inflammatory cytokines IL1-ß, IL-6 and TNFα [10], [11]; latter was also linked to the maintenance of infected cell proliferation in *T. parva*-infected B cells through NF-κB activation [12]. TNFα originally identified as a mediator of inflammatory responses with cytotoxic functions, was recognized more recently as a potential inducer of cancer progression by promoting growth and metastatic dissemination of cancer cells in autocrine and paracrine manner [13]. The capability of TNFα to promote dissemination of epithelial tumor cells has been recognized long ago [14]. However, how the underlying molecular mechanisms such as altered integrin αv β3 expression via IκB induction [15] or FAN (factor associated with neutral sphingomyelinase activity)-induced actin reorganization via Cdc42 [16], [17] affect invasive motility is only poorly understood.

TNFα signals through TNF-R1 and TNF-R2 to induce both pro-apoptotic as well as pro-survival signaling and to generally induce inflammatory responses through JNK and other MAP kinase pathways as wells as NF-κB [13]. TNFα induces the Ste-20 germinal center kinase MAP4K4 (MAP4K4: Mitogen-activated kinase kinase kinase kinase 4, synonyms: HGK: Hepatocyte progenitor kinase-like/Germinal centre kinase-like kinase, NIK: Nck-interacting kinase (murine) [18], which can activate the c-jun N-terminal kinase (JNK) signaling pathway [19], [20]. In Drosophila, the fly ortholog of MAP4K4 misshapen (msn) is activated downstream of TNFα through TNF receptor-associated factor 2 (TRAF2) [21]. More recently, functions of MAP4K4 downstream of TNFα to control systemic processes such as inflammation [22] or insulin resistance [23]–[26] were described. In parallel, unrelated studies a still unclear picture of MAP4K4 functions in cancer progression began to emerge, where MAP4K4 was identified in genetic interference screens as promoter of cell motility and invasiveness [27]–[31]. These studies are supported by findings from *Drosophila* [32]–[36] or *C. elegans* [37]–[39], which linked function of the respective orthologs msn and MIG-15 to cellular processes controlling coordinate cell movements.

We recently found that host MAP4K4 accumulated at the leading edge of polarized, matrix-invading *T. annulata* infected host cells [40]. Since MAP4K4 controls inflammatory signaling pathways downstream of TNFα and cancer cell motility, we investigated MAP4K4 functions in macrophages infected with *T. annulata*, which display a parasite-dependent chronic increase in TNFα expression. We determined the relevance of TNFα signaling for host cell migration and revealed MAP4K4 functions in controlling parasite-dependent actin dynamics regulation and invasive cell motility.

## Results

### TNFα promotes host cell motility and invasiveness in *T. annulata*-infected cells

**Figure 1:**
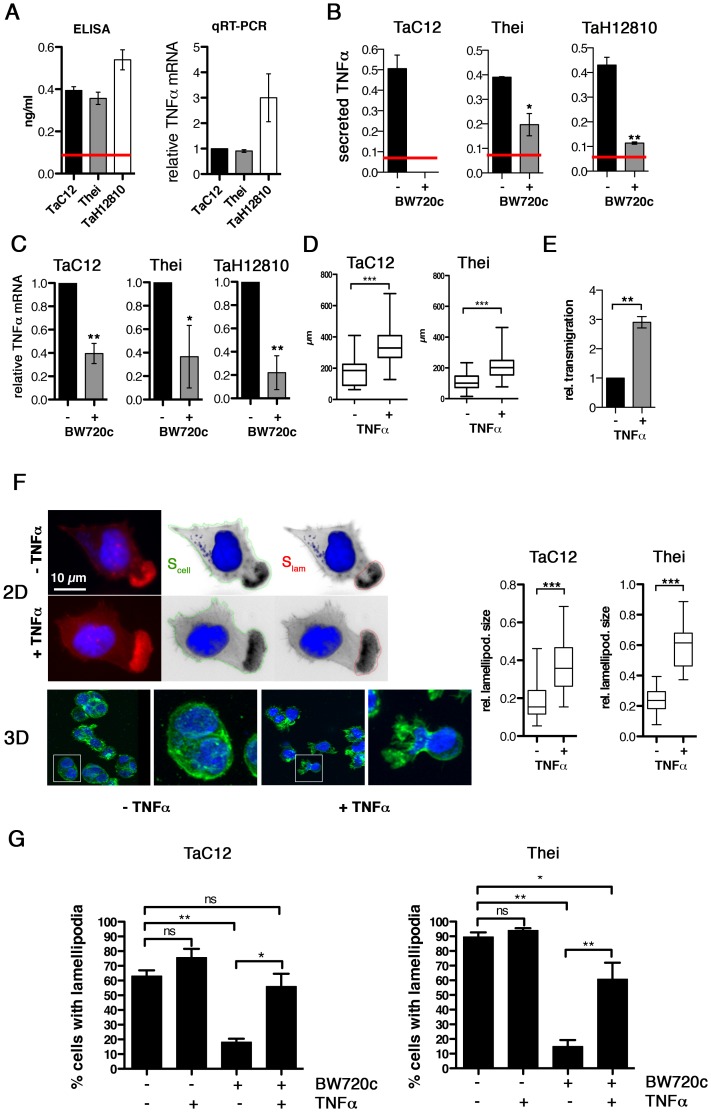
TNFα promotes motility and lamellipodia dynamics of infected macrophages. **A**) Left: Quantification of TNFα concentration in culture supernatants of TaC12, Thei and TaH12810 cells by ELISA. Red line: detection limit (0.078 ng/ml). Right: Quantification of TNFα mRNA expression in TaC12, Thei and TaH12810 cells by qRT-PCR. **B**). Quantification of TNFα concentration in culture supernatants of parasitized or BW720c-cured TaC12, Thei and TaH12810 cells by ELISA. **C**) Quantification of TNFα mRNA expression in parasitized or BW720c-cured TaC12, Thei and TaH12810 cells by qRT-PCR. **D**) Quantification of path lengths of individual TaC12 and Thei cells migrating for 6 and 12 h, respectively, −/+ TNFα (25 ng/ml, TaC12 n = 31, 40, Thei n = 90, 90). **E**) Matrigel invasion assay: Quantification of TNFα-induced transmigration of TaC12 cells in 24 h relative to unstimulated controls. **F**) Left IF images: Visualization of TNFα (25 ng/ml, 24 h) effects on F-actin-rich protrusions in 2D (upper) and inside matrigel (3D, lower). Magnifications are 4× of boxed areas. Right, box plots: Means of relative areas (ratio lamellipodia area∶cell area) of lamellipodia in TaC12 and Thei cells that were quantified as depicted in F upper (TaC12: n = 77, 38, Thei: n = 30, 30). **G**) Mean percentage of cells with lamellipodia in TaC12 and Thei cells treated with BW720c and TNFα as indicated. See also Figure S1.

Previous studies demonstrated a global increase of TNFα in animals parasitized with *Theileria* [11] and increased TNFα expression in infected macrophages derived both from susceptible and resistant animals [10]. Therefore, we quantified TNFα expression in the three different *T. annulata*-infected macrophage cell lines TaC12, Thei and TaH12810 by ELISA and qRT-PCR (Fig. 1A). BW720c treatment eliminates parasite within 48 hours and results in populations of parasite-free, viable cells of isogenic background (not shown). In these “cured” populations, TNFα secretion was significantly reduced in the Thei and TaH12810 lines and below the ELISA kit's detection limit in the TaC12 line (Fig. 1B). Consistently, TNFα mRNA levels in these cell lines were reduced by 60% to 80% parasite elimination compared to untreated control (Fig. 1C). We observed a similar reduction of TNFα expression after pharmacologcial inhibition of NF-κB (not shown), indicating that permanent induction of NF-κB observed in *Theileria*-infected cells [2], [41] is necessary for increased TNFα expression. To determine whether TNFα induced motile cell behavior, we treated the adherent TaC12 or Thei cells with recombinant TNFα, recorded individual cell movements by time-lapse video microscopy and tracked cell trajectories (paths). We plotted cell paths (Fig. S1A) and measured their lengths (Fig. 1D). We found that TNFα treatment significantly increased path lengths, which proportionally reflect cell speeds. Interestingly, TNFα treatment did not affect directionality of migration as neither the directional migration index (distance/path length, DMI, Fig. S1B), nor the average angular deviations of the axis nucleus-leading edge (Fig. S1C) changed. Hence we concluded that increased cell displacement after TNFα treatment is the result of increased speed and not of increased directional persistence of migration. We then compared matrigel invasion of control or TNFα-stimulated TaC12 cells using matrigel-coated Boyden chambers (Fig. S1D and 1E). We found that TNFα-stimulated cells had a significantly higher capability to transmigrate, indicating that TNFα promoted invasive capabilities of *T. annulata-*infected cells.

Lamellipodia are the driving structures for infected cell movements in 2D [4]. Therefore, we compared actin dynamics in lamellipodia of control or TNFα-treated cells by fluorescence live cell imaging using lifeact-EGFP (LA-EGFP). We detected no quantifiable differences in actin dynamics between untreated and TNFα-treated cells (not shown). However, when we determined the size of lamellipodia relative to the size of the whole cell (Fig. 1F, left), we found that TNFα promoted a significant increase in lamellipodium size in TaC12 and Thei cells (Fig. 1F, right), while overall cell size was not affected (S1E). More importantly, cells embedded in matrigel developed invasive protrusions (Fig. 1F, “3D”) reminiscent of those observed in the recently isolated and virulent strain TaH12810 [40]. To determine whether TNFα treatment only stabilizes lamellipodia or also triggers their assembly, we investigated the impact of TNFα stimulation on lamellipodium formation in BW720c-treated cells, which lack lamellipodia. Comparable to the Thei macrophages investigated in our previous study [4] and Fig. 1G, right, the number of TaC12 cells with a single lamellipodium was drastically reduced after parasite elimination (Fig. 1G). TNFα stimulation of cured TaC12 or Thei cells rescued single lamellipodia assembly, strongly indicating that TNFα meditates motility and invasiveness through the control of lamellipodium assembly and stabilization.

Taken together, we found that TNFα production and secretion is parasite-dependent and that TNFα promotes motility and invasiveness of *T. annulata*-infected host cells, likely due to the capability of TNFα to enhance F-actin-rich leading edge protrusions.

### Secreted TNFα is necessary for efficient motility and invasiveness of infected cells

**Figure 2:**
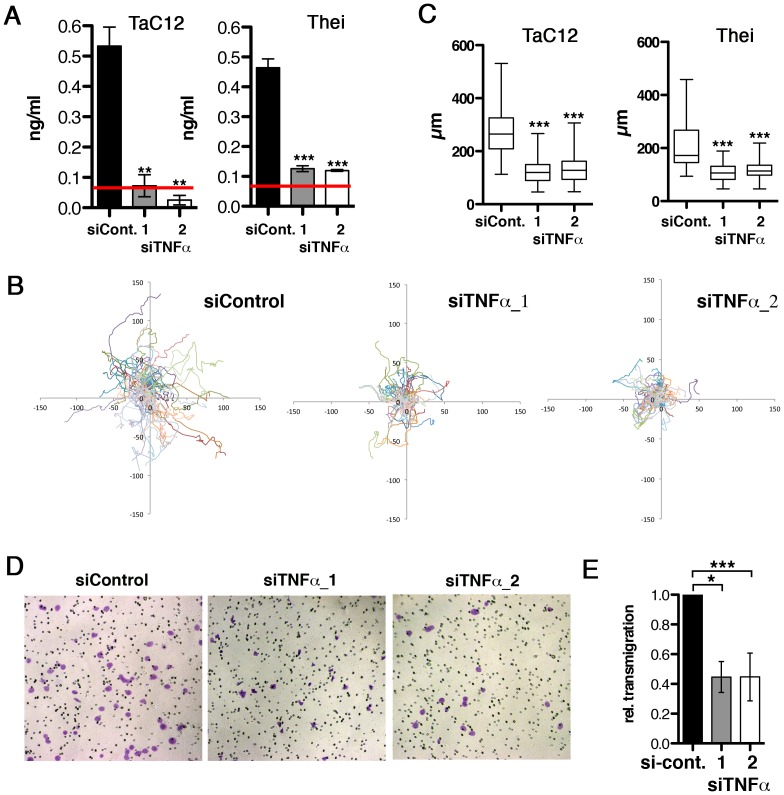
Host cell-secreted TNFα is required for parasite-induced motility. **A**) Quantification of TNFα depletion in TaC12 and Thei culture supernatants by ELISA, 48 h after siControl or siTNFα transfections. Red line: detection limit (0.078 ng/ml). **B**) Paths of individual siControl or siTNFα-transfected TaC12 cells migrating for 8 h. **C**) Quantifications of path lengths of control and TNFα-depleted TaC12 and Thei cells. Box plots with means of path lengths are shown (TaC12 n = 88, 95, 105 and Thei n = 35, 35, 35) **D**) Matrigel invasion assay: Bright field microscopy images (100× magnification) of either siControl or siTNFα_1 or siTNFα_2 transfected cells after transmigration. **E**) Quantification of secreted TNFα dependent transmigration in matrigel assay after 24 h. See also Figure S2.

To determine whether endogenous TNFα is sufficient to promote motility and invasiveness, we silenced TNFα expression in TaC12 cells by two different siRNAs. We effectively reduced the quantity of soluble TNFα protein in the culture supernatants (Fig. 2A), although TNFα mRNA was only moderately reduced (Fig. S2A). Under conditions of reduced soluble TNFα, motility (path lengths) of infected Tac12 and Thei cells was reduced by 60% (Fig. 2B & C) and lamellipodium formation impaired (Fig. S2D). Silencing endogenous TNFα had no effect on directionality of migration (Fig. S2). We next tested whether reduced endogenous TNFα would reduce matrigel invasion, conversely to increased invasiveness after ectopic addition of TNFα. Indeed, decreasing TNFα significantly reduced matrigel invasive and transmigratory capability of TaC12 cells (Fig. 2D &E). Thus, secreted TNFα promotes infected cell motility by increasing the speed of cell migration and by increasing the capability of cells to penetrate stiff matrices. We conclude that *T. annulata*-induced TNFα alters motile cell behavior in a way that facilitates dissemination of infected cells, while the source of TNFα can be both autocrine and paracrine.

### MAP4K4 is constitutively induced and activated in infected cells

**Figure 3:**
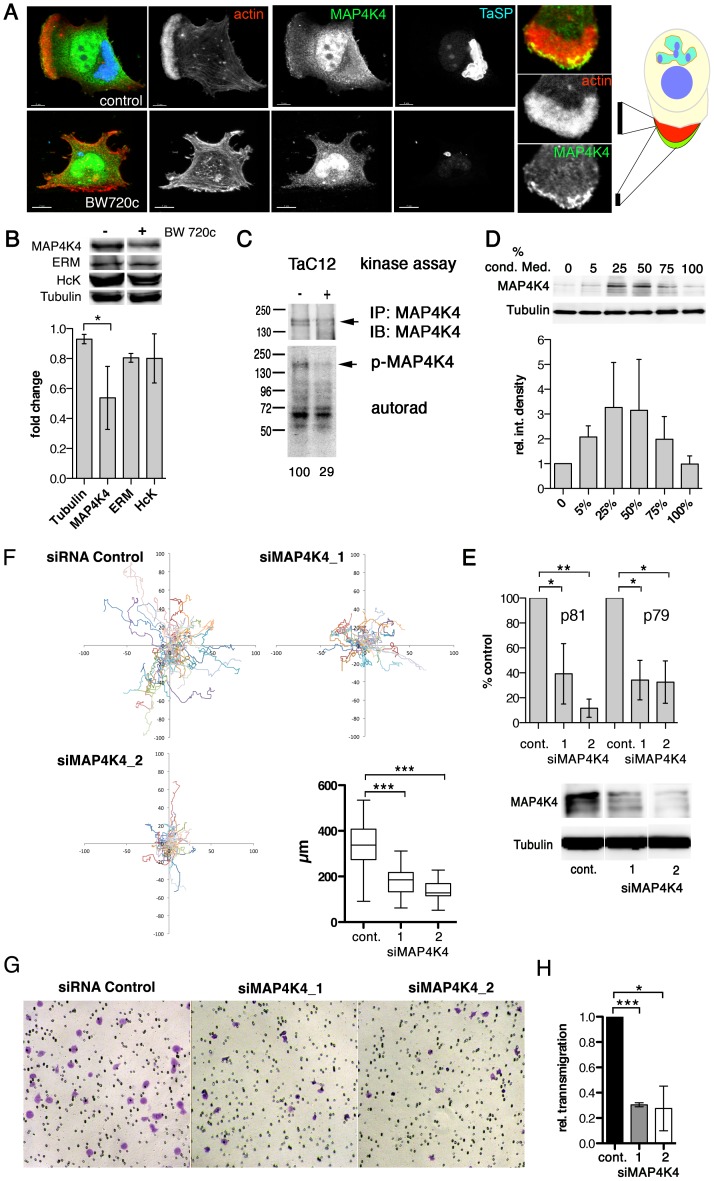
MAP4K4 promotes host cell motility and TNFα-induced invasiveness. **A**) Confocal IFA of intracellular localization of MAP4K4 in parasitized (upper) and BW720c-cured (lower) TaC12 cells. Red: actin, green: MAP4K4, blue: TaSP. Right: magnification of TaC12 lamellipodium showing accumulation of MAP4K4 at leading edge. Schema shows localization of MAP4K4 at leading edge of cell. **B**) IB analysis of untreated and BW720c-treated TaC12 cells using anti-MAP4K4, anti-ERM, anti-Hck and anti-tubulin antibodies. Bar diagram below shows mean protein abundances −/+ SDs. in control and BW720c-treated TaC12 cells. **C**) *In vitro* kinase assay comparing MAP4K4 immunoprecipitated either from infected or cured TaC12 cells. Upper: IB of immunoprecipitated MAP4K4, lower: autorad of total protein phosphorylation activities immunoprecipitated. **D**) Upper: IB analysis with anti-MAP4K4 and anti-tubulin antibodies using lysates of TaC12 cells previously incubated with increasing concentrations of conditioned medium. Lower: Mean integrated densities of MAP4K4 bands (−/+ SDs, normalized to tubulin) **E**) Quantification of MAP4K4 depletion by qRT-PCR using primers p81 and p79 (upper, see also Fig. 5A) or IB (lower) 24 h after siControl or siMAP4K4 transfection. **F**) 12 h single cell migration analysis of cells transfected with either siControl or siMAP4K4_1 or siMAP4K4_2. Box plot show means of path lengths (n = 34, 38, 44). **G**) Matrigel invasion assay: Bright field microscopy images (100× magnification) of transmigrated siControl or siMAP4K4_1 or siMAP4K4_2 transfected cells after 24 h in the presence of 25 ng/ml TNFα. **H**) Quantification of images shown in G expressed as relative transmigration compared to siControl and SDs. See also Figures S3 and S4.

Given the pro-migratory effects of TNFα on *T. annulata*-infected macrophages, we searched for a downstream effector controlling actin dynamics and cell motility. MAP4K4 is TNFα-induced kinase, signaling through the JNK pathway [19], [24] and contributing to migration and metastasis of cancer cells [27]–[29]. In *T. annulata*-infected macrophages, we detected MAP4K4 in the nucleus and in leading edge lamellipodia (Fig. 3A). We next determined whether MAP4K4 protein expression is parasite-dependent by comparing MAP4K4 protein abundance in parasitized and BW720c drug-cured cells by immuno blotting (IB). Fig. 3B upper shows a representative IB with antibodies against MAP4K4, the ezrin, radixin, moesin (ERM) proteins, the Src kinase Hck and tubulin. Average expression levels indicated a moderate (50%) reduction of MAP4K4 protein abundance in cured cells (Fig. 3B, lower). We confirmed moderate MAP4K4 down regulation after parasite elimination in the Thei (Fig. S3A) and TaH12810 (Fig. S3B) lines. Parasite elimination also reduced autophosphorylation capabilities of immunoprecipitated MAP4K4 (Fig. 3C) and its activity towards the substrate MBP *in vitro* (Fig. S3C), indicating that chronic infection of macrophages by *T. annulata* increases MAP4K4 expression and activity. To test whether a secreted host cell factor promoted MAP4K4 expression, we treated TaC12 cells with conditioned medium. 24 h later, we monitored alterations in MAP4K4 expression by IB. Interestingly, conditioned medium promoted increased MAP4K4 expression with maximal effects observed at 50% conditioning (Fig. 3D), suggesting that MAP4K4 expression is induced in an autocrine manner by a parasite-dependent, host cell secreted factor.

### MAP4K4 promotes motility and invasiveness of infected cells

MAP4K4 was identified as a pro-migratory kinase in carcinoma cells [27]. Therefore, we measured whether siRNA-mediated silencing of MAP4K4 would impair infected cell motility. We tested three different MAP4K4-specific siRNAs, from which two (siMAP4K4_1 and siMAP4K4_2) effectively reduced MAP4K4 expression, both at the mRNA and protein levels (Fig. 3E). We quantified path lengths and directionalities of siControl and siMAP4K4 cells by live-cell imaging. Silencing MAP4K4 significantly reduced path length over time (speed) (Fig. 3F), while it did not affect directionality of migration (Fig. S4A & B). Consistently, EGFP-fused wild-type (wt, movies S1 & S2) but not kinase dead (k/d, movies S3 & S4) MAP4K4 promoted a motile phenotype when ectopically expressed in TaC12 cells. We next compared the capability of siMAP4K4 cells to cross matrigel-coated Boyden chambers and found that matrigel invasion was significantly reduced when MAP4K4 was depleted (not shown). Importantly, MAP4K4 silencing also blocked TNFα-induced F-actin-rich cell protrusions (Fig. S4 C & D) and matrigel invasiveness (Fig. 3G & H). These data demonstrate the pro-migratory function of MAP4K4 in infected cells and indicate MAP4K4 function downstream of TNFα to promote invasive cell dissemination.

### TNFα induces MAP4K4 expression

**Figure 4:**
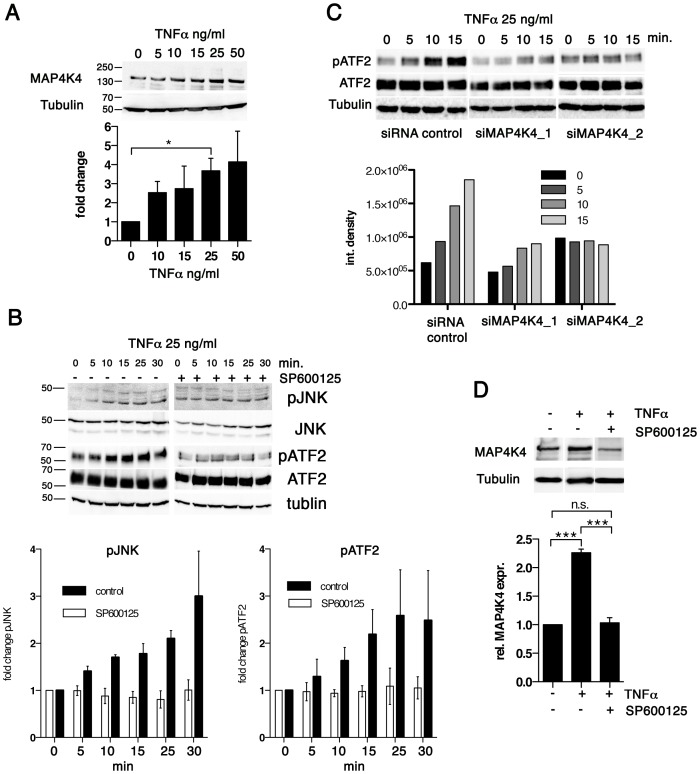
TNFα promotes increased MAP4K4 protein expression through the JNK pathway. **A**) IB analysis of cells treated for 24 h with increasing concentrations of TNFα using antibodies against MAP4K4 or tubulin; Below: quantification of integrated densities of bands (means −/+ SDs, normalized to tubulin). **B**) Time course IB analysis of lysates of cells treated with 25 ng/ml TNFα using antibodies against proteins indicated. Right: IB analysis of effect of JNK inhibitor SP600125 (10 ng/ml, added 2 h before TNFα treatment) on TNFα-induced JNK pathway activation. Bar diagram: Means and SDs of pJNK and pATF2 after TNFα stimulation (normalized to total JNK and total ATF2 proteins, respectively). **C**) Time course IB analysis of lysates of cells treated with 25 ng/ml TNFα using antibodies against proteins indicated. Cells were stimulated 24 h after transfection with siControl or siMAP4K4_1 or siMAP4K4_2. Bar diagram below shows quantification of pATF2 bands (normalized to total ATF2 protein). **D**) IB analysis of lysates from cells treated for 24 h−/+25 ng/ml TNFα and −/+10 ng/ml SP600125, using anti-MAP4K4 or anti-tubulin antibodies. Bar diagram below shows quantification of MAP4K4 bands (means −/+ SDs, normalized to tubulin).

To determine whether TNFα induced MAP4K4 expression, we examined alterations in MAP4K4 expression by IB in TaC12 cells treated with recombinant TNFα. We found that TNFα up-regulated MAP4K4 proteins in a dose dependent manner, with a 3.5 fold increase at 25 ng/ml concentration (Fig. 4A). We also tested whether the two unrelated cytokines HGF, which could induce MAP4K4 in a heterologous system [42] and GM-CSF, which promotes proliferation of *Theileria* infected cells [43] affected MAP4K4 expression. Neither HGF nor GM-CSF significantly increased MAP4K4 expression within 24 hours of treatment (not shown). TNFα activates the JNK signaling pathway [20], which is constitutively activated in different *Theileria*-infected cell lines [44]–[46]. Consistently, phosphorylation of JNK on Thr183 and Tyr185 and of its downstream substrate ATF2 on Thr71 in TaC12 cells is increased in a time-dependent manner after TNFα treatment (Fig. 4B). Pre-treatment with the JNK inhibitor SP600125 prevented TNFα-induced JNK phosphorylation and activation ATF2 (Fig. 4B, right). We next silenced MAP4K4 before TNFα treatment and found that MAP4K4 depletion markedly reduced TNFα-induced activation of the JNK substrate ATF2 (Fig. 4C), indicating MAP4K4 functional relevance in TNFα-dependent JNK pathway activation in *T. annulata*-infected cells. Interestingly, JNK signaling was needed for TNFα-induced MAP4K4 expression because the stimulatory effect of TNFα on MAP4K4 expression was inhibited when JNK activity was pharmacologically blocked (Fig. 4D).

Thus, TNFα contributes to JNK signaling in infected cells and promotes MAP4K4 expression most likely via JNK-mediated ATF2 activation [19], while MAP4K4 in turn is needed for TNFα-induced JNK activation. Hence, the MAP4K4-JNK signaling module is active in infected cells and integrates TNFα signaling into MAP4K4 induction in a positive feedback type mechanism.

### MAP4K4 expression is regulated at transcriptional and post-transcriptional levels

**Figure 5:**
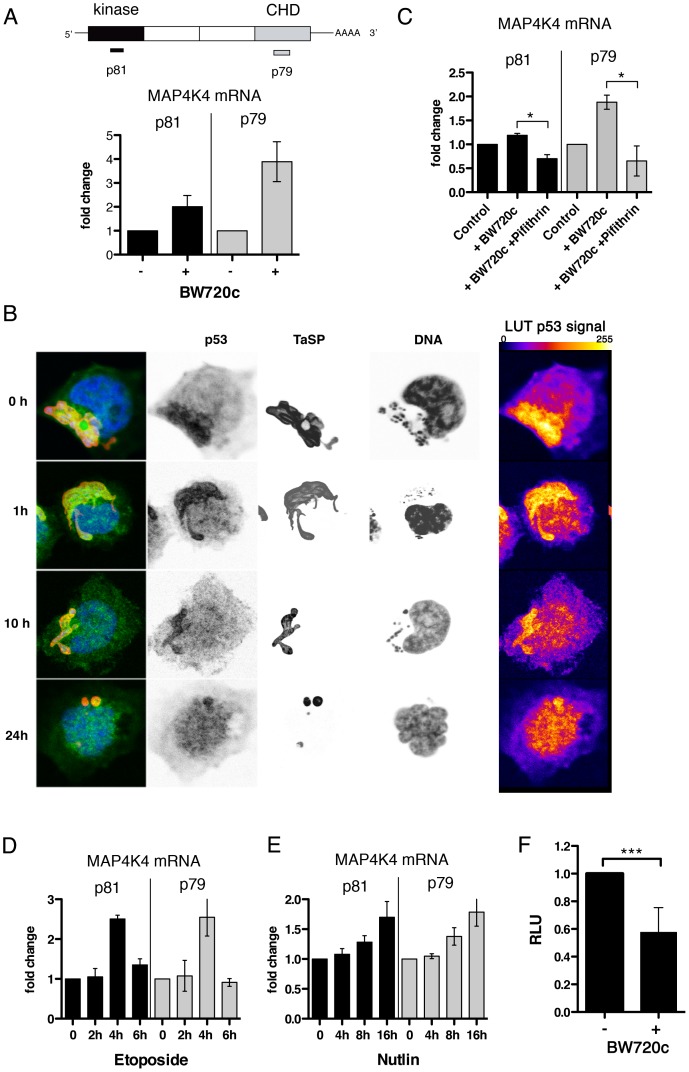
MAP4K4 mRNA expression level is parasite dependent. **A**) Quantification of MAP4K4 mRNA expression levels by qRT-PCR. GAPDH mRNA was used as a reference. Expression levels after BW720c relative to untreated control −/+ SDs are shown. Schema shows relative localization of qRT-PCR primer binding sites. **B**) Confocal IFA of p53 (anti-p53, green), TaSP (TaSP, red) and DNA (hoechst, blue) localization in infected macrophages and after increasing durations of BW720c treatment. Look-up tables (LUTs) of p53 signal shows 255 grey scale images of p53 fluorescence, displayed as fire LUTs. **C**) mRNA expression analysis of MAP4K4 by qRT-PCR after treatment with BW720c alone or together with Pifithrin (5 µg/ml = 13.6 µM). mRNA expression relative to untreated control ± SDs is shown. **D**) Quantification of etoposide (25 µg/ml = 42 µM) treatment effects on MAP4K4 mRNA expression by qRT-PCR. Time course expressions relative to untreated control ± SDs are shown. **E**) Quantification of Nutlin-3 (5 µM) treatment effects on MAP4K4 mRNA expression by qRT-PCR. Time course expressions relative to untreated control ± SDs are shown. **F**) Comparison of luciferase-MAP4K4-3′UTR activities in control and BW720c-treated cells. Normalization with luciferase-GAPDH-3′UTR. See also Figure S5.

Interestingly, qRT-PCR analysis revealed strikingly higher MAP4K4 mRNA levels in cured cells (Fig. 5A). Therefore, we hypothesized that MAP4K4 expression could be transcriptionally up-regulated after parasite elimination. The only transcription factor described so far besides ATF2 [19] regulating MAP4K4 is p53 [47]. *T. annulata* was recently shown to sequester host cell p53 in infected cells [48]. Indeed, in TaC12 cells, p53 gradually translocated from the parasite surface to the host cell nucleus after BW720c treatment (Fig. 5B). Therefore, we blocked p53 function in cured cells with Pifithrin [49] and quantified MAP4K4 mRNA expression by qRT-PCR. We found that BW720c-induced MAP4K4 was completely abrogated by Pifithrin (Fig. 5C), suggesting that p53 promotes increased MAP4K4 mRNA induction in drug-cured cells. Conversely, when we triggered p53 function in parasitized cells, either by induction (etoposide, Fig. 5D) or stabilization (Nutlin-3, Fig. 5E), we observed a time-dependent increase in MAP4K4 expression. Both compounds led to the accumulation of p53 in the nucleus of infected cells, indicative for p53 activation (Fig. S5A). Moderate proteasome-mediated MAP4K4 degradation after BW720c treatment (Fig. S5B) cannot fully account for reduced MAP4K4 protein in the presence of high levels of MAP4K4 mRNA. We alternatively hypothesized that MAP4K4 mRNA is translationally blocked by miRNA binding to the 3′ UTR of the MAP4K4 mRNA. Using TaC12 cells stably expressing luciferase fused to MAP4K4 3′UTR or to GAPDH 3′UTR as control, we found that parasite elimination significantly and specifically reduced luciferase-MAP4K4 3′UTR expression, suggesting that the presence of *T. annulata* in the host macrophage repressed miRNA targeting of the 3′UTR of MAP4K4 (Fig. 5F). Together, our data show that increased MAP4K4 mRNA after parasite elimination requires functional host cell p53 induction and indicate miRNA-mediated translational repression of newly synthesized MAP4K4 mRNA.

### Regulation of lamellipodia dynamics by MAP4K4 involves ERM protein activation

**Figure 6:**
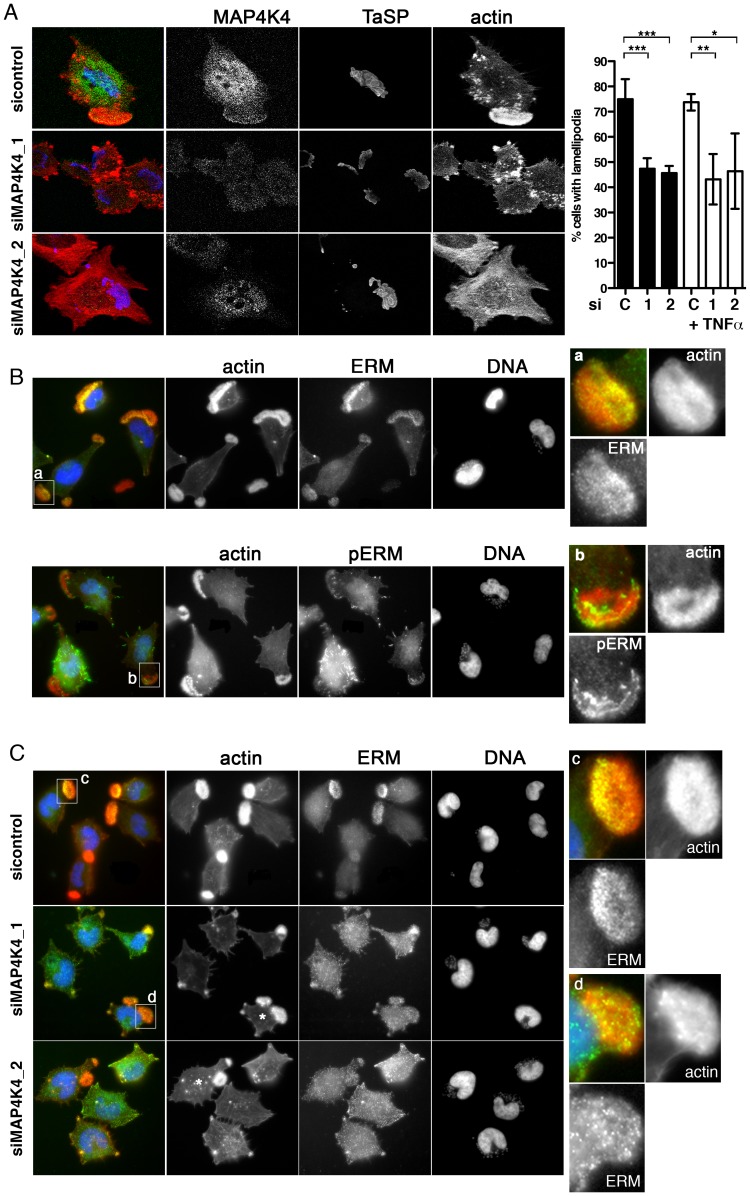
MAP4K4 promotes lamellipodium formation and enhances ERM accumulation in lamellipodia. **A**) Confocal IFA of endogenous MAP4K4 (green), F-actin (red) and TaSP (blue) in cells transfected with siControl or siMAP4K4. Bar diagram shows quantification of percent cells displaying a large, single lamellipodium in the absence (black) or presence (white) of 25 ng/ml TNFα. C: siControl; 1: siMAP4K4_1; 2: siMAP4K4_2 **B**) IFA of total ERM (anti-ERM ab) proteins and ERM proteins phosphorylated on C-terminal threonine (anti-pERM ab) in cells seeded on Fn. **a** and **b** show 4× magnifications of boxed areas for ERM (**a**) and pERM (**b**) distribution. F-actin is in red, ERM and pERM in green, DNA in blue. **C**) IFA of ERM localization in siControl or siMAP4K4 transfected cells. IFA as in B, **c** and **d** show 4× magnifications of boxed areas for ERM accumulation in lamellipodia of siControl (**c**) and or siMAP4K4 transfected cells (**d**). See also Figure S6.

**Figure 7:**
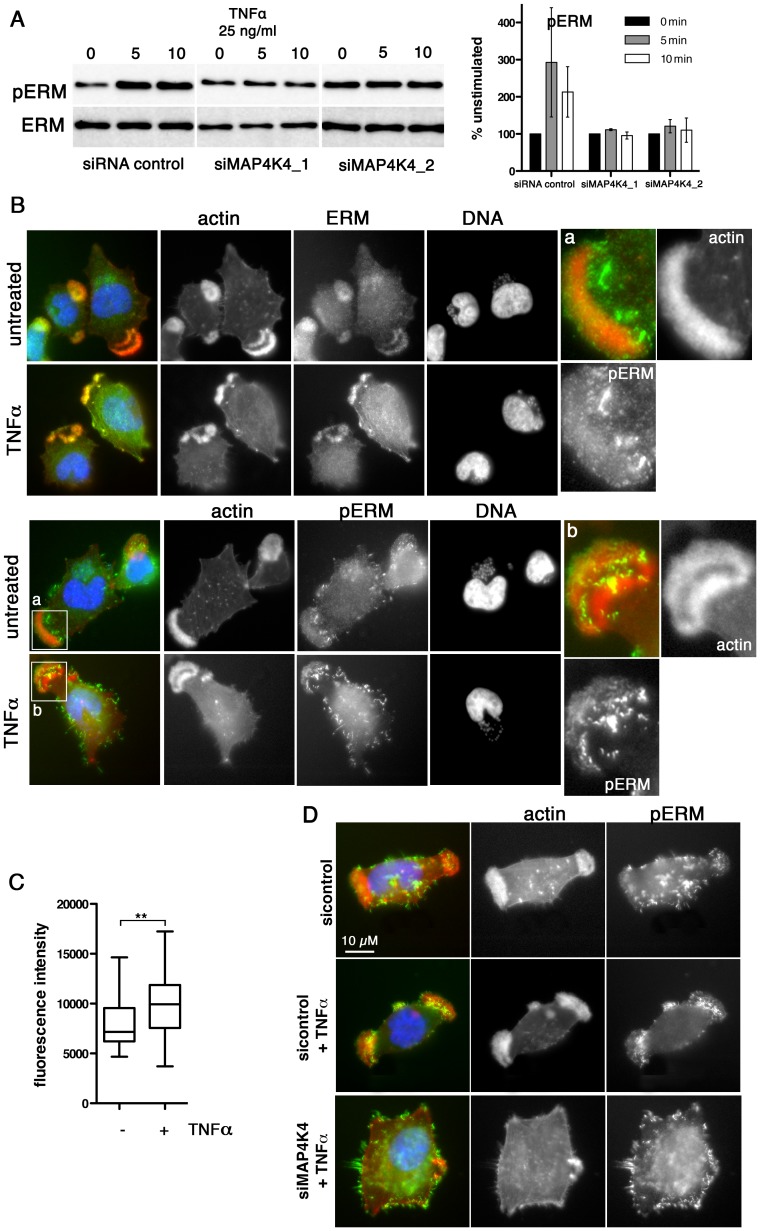
TNFα-induced ERM protein activation is MAP4K4 dependent. **A**) IB analysis of TNFα-induced ERM protein phosphorylation using anti-ERM and anti-pERM antibodies in lysates of cells transfected with siControl or siMAP4K4_1 or siMAP4K4_2. Representative IB and quantifications are shown. **B**) IFA of total ERM proteins (ERM) and pERM proteins (pERM) in cells seeded on Fn. **a** and **b** show 4× magnifications of boxed areas for pERM distribution in lamellipodia of untreated (**a**) or TNFα-treated cells (**b**). F-actin is in red, ERM and pERM in green, DNA is blue. **C**) Quantification of pERM fluorescence intensities in lamellipodia of unstimulated (−) and TNFα-stimulated (+) cells (n = 34, 34). **D**) IFA of F-actin and pERM proteins in siRNA-transfected cells seeded on Fn. F-actin is red, pERM in green, DNA blue. See also Figure S6.

Approximately 70–80% of TaC12 cells harbor one to three large, F-actin-rich lamellipodia (Fig. 1F, 3A & 6A). Silencing MAP4K4 reduced this number to 47 and 46% for siMAP4K4_1 and siMAP4K4_2, respectively (Fig. 6A), indicating MAP4K4 implication in lamellipodium assembly. The membrane-F-actin cross-linker proteins of the ezrin, radixin and moesin (ERM) family accumulate in persistent lamellipodia of *T. annulata*-infected macrophages [4] whereas MAP4K4 phosphorylates ERM proteins on the regulatory C-terminal threonine residue to control lamellipodia dynamics [50]. Therefore, we tested by IFA as to whether MAP4K4 could control ERM protein accumulation in lamellipodia of *T. annulata*-infected cells. In control siRNA transfected cells, ERM proteins accumulate throughout lamellipodia, while activated (phosphorylated) ERM (pERM) proteins localize more distally towards the leading edge (Fig. 6B), where also MAP4K4 localizes (Figs. 3A & 6A). MAP4K4 depletion markedly reduced lamellipodia formation and, consequently, also ERM protein accumulation in lamellipodia (Fig. 6C). Reduced ERM accumulation under siMAP4K4-treated conditions was also evident in cells that displayed F-actin-rich lamellipodia (Fig. 6C, compare c and d). In MAP4K4 depleted cells, pERM was detectable in lateral and basal filamentous protrusions when no lamellipodia were present, possibly representing MAP4K4-independent ERM protein phosphorylation (Fig. S6) also detected by IB (Fig. 7A). Interestingly, TNFα treatment led to a marked global increase in C-terminal ERM phosphorylation (Fig. 7A). In MAP4K4 depleted cells, TNFα-induced ERM phosphorylation was no longer evident. This indicated that it was MAP4K4 that mediated ERM protein phosphorylation downstream of TNFα, while another kinase must contribute to the maintenance of the global pERM levels in unstimulated cells, because non TNFα-induced pERM was not reduced in MAP4K4-depleted cells. This observation is consistent with the findings of our IFA analyses (Fig. S6), where lammellipodia-localized but not global pERM was altered. In lamellipodia of TNFα-treated cells, we observed focal accumulations of pERM proteins (Fig. 7B). We compared fluorescent intensities of pERM in lamellipodia of unstimulated and TNFα-stimulated cells and quantified a moderate but significant 24% increased in pERM in stimulated cells (Fig. 7C). However, TNFα treatment did not rescue lamellipodia formation in cells where MAP4K4 was previously silenced (Fig. 6a & 7D), corroborating the notion that TNFα is upstream of MAP4K4 regulation of lamellipodia dynamics. Together, our data show that TNFα induces ERM phosphorylation on the regulatory C-terminal threonine in a MAP4K4-dependent manner and that this ERM phosphorylation likely occurs in lamellipodia.

## Discussion

In this manuscript we established TNFα-dependent induction of host MAP4K4 as a novel mechanism triggered by the intracellular presence of the parasite *T. annulata* to promote dissemination of its host cell. We identified MAP4K4 as a critical intermediate that bifurcates TNFα signaling to JNK pathway activation on one hand and to the activation of the ERM family of cytoskeleton regulatory proteins and actin dynamics on the other hand. JNK pathway activation ensures MAP4K4 expression, while ERM activation spatio-temporally correlated with persistent lamellipodia formation and invasive cell motility (schema Fig. S7). We propose MAP4K4 as a novel effector kinase linking TNFα signals to invasive cell motility regulation under conditions of chronic exposure to TNFα, such as during pathogen infections, in inflammatory disorders and in cancerous lesions.

Several groups previously reported chronic TNFα induction in *Theileria*-infected cells. However, since TNFα expression levels were comparable in *ex vivo* cultures derived from *T. annulata*-infected cattle breads of different disease susceptibilities, its impact as host virulence factor was not further investigated [10]. Guergnon *et al.* linked TNFα expression to NF-κB induction and infected cell proliferation [12], consistent with studies in other systems demonstrating TNFα impact on pro- and anti-apoptotic signaling and its role in inflammation regulation [13]. Here, by characterizing its role in promoting cell motility, we revealed an additional function of TNFα that is largely independent of its role in proliferation/survival regulation. By demonstrating a mechanistic link of TNFα signaling to the established motility regulator MAP4K4 [27]–[31], we provide an explanation for how TNFα expression contributes to Tropical Theileriosis pathogenesis and how TNFα production could be linked with the progression of tumors to invasive cancers [13]. We found that a secreted factor controls MAP4K4 expression because conditioned medium derived of infected cells promoted the expression of MAP4K4. This effect was phenocopied by the addition of recombinant TNFα to unconditioned, fresh medium. Optimal MAP4K4 induction was achieved at 50% conditioning (rather than at 100%), possibly either due to nutrient starvation in media with a higher ratio of conditioned medium or due to an MAP4K4 inhibitory factor secreted by cells grown to confluence. Unlike TGFβ, who's expression is increased in infected cells derived from susceptible host animal [3], TNFα expression was found to be similar in cattle breeds with different disease susceptibilities [51]. Hence, TNFα and TGFβ action could synergize to promote host cell virulence, whereby TNFα enables primary motile properties that are further enhanced after exposure to TGFβ. Importantly, TGFβ controls the expression of additional pro-migratory factors including hepatocyte growth factor (HGF) [3], which can promote motility of *Theileria*-infected cells without affecting MAP4K4 expression (Ma and Baumgartner, unpublished observation). Thus, *Theileria*-infected cells produce a mixture of factors including TGFβ, HGF and TNFα that contribute individually and collectively to cell motility, whereby TGFβ and TNFα are coupled directly to cell motility regulation through Rho-kinase ROCK [3] and MAP4K4 (herein), respectively.

We propose the promigratory effect of MAP4K4 to be due to its capability to affect actin dynamics in cellular protrusions by direct C-terminal phosphorylation of ERM family proteins [50], and possibly also by alternative mechanisms including direct phosphorylation of actin modulatory proteins [52]. Indeed, ERM protein activation is associated with parasite-dependent host cell polarization [40], [53], whereas it is also considered an important event during cancer metastasis [54], [55]. Although it is still not entirely clear how similar the processes are that drive invasiveness of *Theileria*-infected cells and cancer cells, polar activation of ERM proteins is a mechanism likely generally conserved in invading cells. Several C-terminal ERM kinases have been proposed as ERM activators (reviewed in [56]) to modulate the cortical actin cytoskeleton and morphodynamic processes [57]. Depletion of MAP4K4 by siRNA completely abrogates TNFα-induced ERM phosphorylation, suggesting that it is mainly MAP4K4 that mediates C-terminal ERM phosphorylation downstream of TNFα in *T. annulata*-infected cells. Using pharmacological approaches, ERM phosphorylation downstream of TNFα was also previously shown and linked to p38 MAPK and PKCs in endothelial cells [58] or p38 MAPK and Rock activities in fibroblast-like syno-viocytes [59]. However, both studies showed ERM phosphorylation kinetics that peaked after one hour and contrast our finding of rapid (within 5 min) ERM phosphorylation, suggesting that immediate ERM phosphorylation by MAP4K4 is direct. Interestingly, a recent study revealed phosphorylation of the ERM protein moesin by JNK for podosome rosette formation in Src-transformed fibroblasts [60]. It is conceivable that an analogous JNK-mediated phosphorylation of ERM proteins may be active as well for parasite-dependent podosome formation in *Theileria*-transformed cells, which requires Src kinase activity too [4], possibly causing residual ERM phosphorylation in MAP4K4-depleted cells. Consistent with the notion of selective, spatially restricted activity of JNK and MAP4K4 towards ERM proteins, we observed no co-localization of MAP4K4 with podosomes in infected cells. ERM activation by MAP4K4 likely structurally stabilizes the interaction of newly polymerized F-actin filaments with membrane proteins, to promote lamellipodia in 2D and invasive protrusions in 3D and to facilitate assembly of signaling complexes. ERM proteins can also act as protein kinase A anchoring proteins to affect cAMP-induced signaling pathways [61], suggesting that TNFα-induced ERM phosphorylation could also affect PKA signaling active in *T. annulata*-infected cells [62].

MAP4K4 expression at the mRNA level increased after parasite elimination while MAP4K4 protein decreased. We explain this conundrum with two unrelated but parasite-dependent mechanisms. First, using either specific inhibitors or activators of p53, we concluded that p53 released from the parasite surface – where it is sequestered when the parasite is intact [48] - can transcriptionally induce MAP4K4. The use of a proteasome inhibitor under these conditions did not indicate that the consequently increased amounts of MAP4K4 protein is diminished by proteasomal degradation; rather our data indicate miRNA-dependent targeting of the MAP4K4 3′UTR to limit MAP4K4 protein expression. Thus, MAP4K4 protein abundance is regulated by JNK-ATF2 signaling in parasitized cells and after parasite elimination, by p53 combined with translational repression by miRNA. Indeed, parasite-dependent regulation of host cell miRNA expression has just recently been demonstrated [63]. miRNA targeting of MAP4K4 could be a mechanism evolved to protect cells from overabundance of this potentially oncogenic kinase.

Together, we highlight the potential functional significance of TNFα signaling in Tropical Theileriosis, and we revealed a pathogen dependent-mechanism controlling oncogene expression and function to promote invasive motility of its host cell. Analogous to *Theileria*-induced host cell transformation, MAP4K4 signaling might be relevant in driving cancer cell dissemination in tumors, where increased levels of anti-cancer therapy-induced TNFα has been noted in the surrounding stroma [64].

## Materials and Methods

### Cell culture

TaC12 cells (strain Ankara [65]) were a gift from Dirk Dobbelaere. TaH12810 cells (line H7, generous gift from Elizabeth Glass, The Roslin Institute, Edinburgh) were established *ex vivo* from the peripheral blood from Holstein calves previously infected with *T. annnulata* Hisar sporozoites [10]. The Thei line is a well-established laboratory line-derived of a naturally infected animal [44], [66] (generous gift from Gordon Langsley). *T. annulata*-infected macrophage lines were cultured in RPMI-1640 (R-0883, SIGMA) with 10% heat inactivated fetal bovine serum (FBS) (10500-064,GIBCO), 1% penicillin/streptomycin and 0.001% ß-Mercaptoethanol (60242, SIGMA-ALDRICH). This medium is refereed to as complete medium. Starvation medium is complete medium with 0.5% FBS. Cells were cultured at 37°C in a humidified atmosphere containing 5% CO_2_. Parasite elimination (“cure”) was done with 50 ng Buparvaquone (BW720c)/ml for 48 h. Conditioned medium cultures: Fresh complete medium was added to 50–60% confluent cells. After 24 h incubation, supernatant (sn) was recovered, centrifuged to remove floating cells, sterile filtered and stored at −80°C. For medium conditioning, increasing volumes of sn were mixed with decreasing volumes (100, 75, 50 and 25%) of fresh medium.

### Reagents

EGFP-NIKwt and EGFP-NIKD152N [50]. pCDNA3B-MAP4K4-wt-Myc-His and pCDNA3B-MAP4K4-K/R-Myc-His [42]. pLENTI-LA-EGFP, pLENTI-LA-mCherry (generous gift of Olivier Pertz, Basel), pYFP-ezrin (generous gift of Miguel Quintanilla, Madrid), pLenti-blank-luc, pLenti-luc-GAPDH-3′UTR and pLenti-luc-MAP4K4-3′UTR (Applied Biological Materials, Richmond, Canada) Etoposide (WI383, SIGMA), Nutlin-3 (10004372, Cayman), MG-132 (Calbiochem CAS133407-82-6), human recombinant TNFα (300-1A, PEPROTECH, 25 ng/ml), SP600125 (S5567, SIGMA), Pifithrin (P4359, SIGMA), Buparvaquone was a gift of Dirk Dobbelaere (Vetsuisse Faculty, Bern).

### Primer and siRNA sequences

#### siRNAs

siTNFα_1: 5′-AGA CAC CAU GAG CAC CAA ATT-3′; siTNFα_2: 5′-GAU CUC ACC UAG AAC UUG ATT-3′; siMAP4K4_1: 5′-UAC CCU UCA CAU CUC AUU ATT-3′; siMAP4K4_2: 5′-CUU GAG CAA UGG UGA AAC ATT-3′;

#### qRT PCR primers

TNFα_F: 5′-CCA CGT TGT AGC CGA CAT CA-3′; TNFα_R: 5′-CTG GTT GTC TTC CAG CTT CAC A-3′; 1(p79)_MAP4K4_F: 5′-TAC CGA GAG TGG CCT GAT GCT-3′; 1(p79)_MAP4K4_R: 5′-CAA CCT CCG GGT CGT TGT GAA G-3′; 2(p81)_MAP4K4_F: 5′-CAT TAG GGA TCA GCC GAA CG-3′; 2(p81)_MAP4K4_R: 5′-GTT GAC GAT GGA GCT GGG CT-3′; GAPDH_F: 5′-GGT GAT GCT GGT GCT GAG TA-3′; GAPDH_R: 5′-TCA TAA GTC CCT CCA CGA TG-3′

### Matrigel 3D cultures

Cells were suspended in RPMI-1640 medium and mixed with growth factor reduced Matrigel (354230, BD BioScience) at a ratio of 1∶9. 10 µl of the cell-matrigel suspension were transfer per well into 15 well μ-Slide (81506, Ibidi). After polymerization of the matrigel, the wells were filled with 50 µl medium (with or without treatment). Cells were fixed by 4% PFA after 24 hours in culture.

### Matrigel invasion assay

For matrigel invasion assays, matrigel-coated Boyden chambers (BD 354480) were used. 25'000 cells were suspended in RPMI (0.5% FBS) and seeded on the upper side of the matrigel-coated membrane; the lower side of the membrane was submerged in complete medium or 0.5% FBS medium supplemented with 25 ng/ml TNFα. After incubation for 24 hours at 37°C, transmigrated cells passing through the membrane were fixed with 4% PFA, stained with 0.05% crystal violet, and viewed and counted using a bright-field microscope.

### Single-cell motility assay

Cells were seeded on 8 well chamber slides (80826, ibidi) at 40% confluency in assay medium with or without TNFα. Time-lapse movies were acquired on an automated Leica LX microscope equipped with a Hamamatsu EM-CCD camera in differential interference contrast (DIC) bright field modus using a 10× dry NA 0.3 objective lens. Cells were maintained at 37°C in a humidified atmosphere containing 5% CO_2_. Cell speed was determined by manually tracking the cells every 5 minutes for 6–12 hours using ImageJ software (NIH image J software).

Directionality of migration index (DMI) in a given time equals the quotient of the rate of displacement divided by the length of the corresponding path, yielding a number between 0 and 1.

Average angular deviations in radians of the axis nucleus-leading edge were determined by assessing the angular deviations of one time point from the corresponding axis of one time point earlier.

### ELISA

TNFα concentrations in cell culture supernatants were measured by ELISA (Bethyl, E11-807) according to manufacturer's instructions. Supernatant samples for TNFα ELISA were collected from cells 24 h after passaging into fresh medium. BW720c-treated cells were treated for 48 h and then re-seeded in fresh medium. si-RNA-transfected cells were re-seeded in fresh medium 24 h after transfection. For both treatments, samples for ELISA were taken 24 h after re-seeding and compared to compared to samples derived from the same number of control cells.

### Immuno blotting (IB)

Total proteins were extracted with modified RIPA lysis buffer (50 mM Tris pH 7.4, 150 mM NaCl, 1 mM EDTA pH 8.0, 1% NP-40, 0.25% NA-deoxycholate, 2 mM Na-vanadate, 5 mM NaF, 1× protease inhibitors (Roche). Proteins were separated by SDS-PAGE gel, transferred onto nitrocellulose membrane (BIO-RAD), probed with primary antibodies followed by HRP-linked secondary antibodies and detected by enhanced chemiluminescence assay (Thermo Scientific). The antibodies used for immunoblotting were: rabbit anti-MAP4K4 (A301-502A, BETHYL, 1∶1000), rabbit anti-ERM (3142, Cell Signaling, 1∶1000), rabbit anti-Phospho-ERM (3149, Cell Signaling, 1∶1000), mouse anti-HcK (610278, BD Biosciences, 1∶1000), mouse anti-beta-Tubulin (T4026, SIGMA-ALDRICH, 1∶10'000), rabbit anti-Phospho-SAPK/JNK (Thr183/Tyr185) (4668, Cell Signaling, 1∶1000), rabbit anti –SAPK/JNK (9258, Cell Signaling, 1∶1000), rabbit anti-Phospho-ATF2 (Thr71) (9221, Cell Signaling, 1∶1000), rabbit anti-ATF2 (9226, Cell Signaling, 1∶1000), secondary anti-mouse and anti-rabbit (7076 and 7074, Cell signaling, 1∶5000).

### Immunofluorescence analyses (IFA)

The cells were fixed with 4% paraformaldehyde (PFA), permeabilized with 0.5% Triton X-100/PBS and blocked with 10% FBS/PBS. The primary antibodies used were: mouse mc anti-MAP4K4 (MO-7, clone 4A5, Abnova 1∶100), rabbit anti-ERM (3142, Cell Signalling, 1∶100), rabbit anti-Phospho-ERM (3149, Cell Signalling, 1∶100), mouse anti-p53 (2524, Cell Signalling, 1∶100). The secondary antibodies used in immunostaining were: Cy3-conjugated Donkey Anti-Rabbit IgG (H+L) (711-165-152, Jackson Immunoresearch, 1∶500), Cy5-conjugated Rat Anti-Mouse IgG (H+L) (415-175-166, Jackson Immunoresearch, 1∶500), Phalloidin, Tetramethylrhodamine B isothiocyante (P1951, SIGMA-ALDRICH, 1∶1000), Texas Red-X phalloidin (T7471, Molecular Probes, 1∶1000), Alexa Fluor 488 anti-mouse IgG, (A11029, Invitrogen, 1∶1000), Alexa Fluor 488 anti-rabbit IgG (A11034, Invitrogen, 1∶1000), Texas Red-X anti-mouse IgG (T862, Invitrogen, 1∶1000), Texas Red-X anti-rabbit IgG (T6391, Invitrogen, 1∶1000), phalloidin-488 (Molecular Probes, 1∶500)

### Actin dynamics

TaC12 cells were infected with pLENTI-LA-EGFP. Actin dynamics were then monitored by live-cell microscopy using a Leica SP5 confocal microscope with temperature and climate control. Images were acquired using a Hamamatsu EM-CCD camera and assembled and analyzed using Imaris software.

### Kinase activity assay

Cells were lysed in RIPA buffer without SDS (under rotation, 4°C, 30 min). Lysates were cleared by centrifugation (10 krpm, 4°C, 10 min). Endogenous MAP4K4 or ectopically expressed MAP4K4-Myc was immunoprecipitated (under rotation, 4°C, 4 h) from 900 µg total protein using 2 µg polyclonal anti-MAP4K4 antibody (Bethyl, 502A) or monoclonal anti-Myc (9E10, Genetex). Ab-MAP4K4 complexes were bound to magnetic beads (millipore, 4°C, 30 min) and washed three times with lysis buffer and once in kinase buffer (25 mM HEPES pH 7.4, 1 mM DTT, 10 mM MgCl2, 3 mM MnCl2). Kinase reaction in 30 µl kinase buffer supplemented with 10 µM cold ATP, 6.25 µCi ^32^PγATP and 2.5 µg/sample MBP 20 min at 30°C. Phosphorylated proteins were revealed by SDS-PAGE followed by autoradiography of dried gels.

### RNA extraction and Real-Time Quantitative PCR

Total RNA was isolated (kit 74106, QIAGEN) and reverse transcribed (Applied Biosystems) using oligodT primers according to manufactures' instructions. qPCR reactions (Applied Biosystems) were performed on an ABI 7900HT apparatus. Primers are listed in paragraph “primer and siRNA sequences”. All quantifications were normalized to control endogenous GAPDH. Relative changes in gene expression were quantified using the 2-^ΔΔC^_T_ methods.

### siRNA transfection

siRNAs were designed and synthesized by Microsynth AG (Balgach, Switzerland). siRNA sequences are listed in paragraph “primer and siRNA sequences”. TaC12 cells were transfected by lipofection according to manufacturer's (11668-019, Invitrogen) instructions. Negative Control siRNA (si-control, 1027280, QIAGEN) was used as a negative control for siRNA transfection.

### Plasmid transfection

Plasmids were transfected using the Amaxa nucleofection protocol with SF solution and program DS103. 0.5×10^6^ cells were used per transfection with 1.5 µg plasmid DNA in 6.5 µl SF solution and 3.6 µl supplement.

### Luciferase 3′UTR assays

pLenti-luc-hsGAPDH-3′UTR and pLenti-luc-hsMAP4K4-3′UTR (Applied Biological Materials, Richmond, Canada) constructs were used to generate lentiviruses. The majority of miRNA target sites in the human MAP4K4 3′UTR predicted to bind specific miRNAs using TargetScan software are also conserved in the bovine MAP4K4 3′UTR (see supporting table S1). Stable TaC12 cell lines that express luciferase under the respective 3′UTR control were generated by puromycin selection. Variation in transgene abundance between GAPDH-3′UTR and MAP4K4 3′UTR in stable lines was determined using qRT-PCR with luciferase-specific primers and used to normalize luciferase measurements. Luciferase activity was determined according to manufacturer's (Promega) instructions.

### Statistical analysis

Statistical analysis and significance tests were performed using Prism5 software. Data are calculated as means ± SDs and expressed either as means of absolute values or as means of changes relative to control treatments. P values were determined using two-tailed paired or unpaired students T-test. Paired T-tests were performed when changes in a pair before-after treatment were compared. Normal distribution of sample values (n≥30) was confirmed when multiple individual measurements were compared (cell tracking, relative lamellipodia sizes, directionality indices). Choice of cells and fields was always random to minimize bias. Box plots show 25th percentile, median and 75th percentile; whiskers show minimal to maximal distributions. At least 3 independent experiments were performed. P values of <0.05 were considered statistically significant. T-tests: * = p<0.05; ** = p<0.001 and *** = p<0.0001.

## Supporting Information

Figure S1 **Exogenous TNFα does not affect cell spreading or directionality of migration.** **A**) Tracks of single TaC12 cells in the absence (control) or presence of exogenous TNFα (25 ng/ml). **B**) Box plots of FMI (ratios of distance/path length) of control and TNFα-stimulated cells (n = 90 cells per group). **C**) Histogram shows frequencies of degrees of angular turns per step of control and TNFα-stimulated cells expressed in radians (n = 90 cells per group). **D**) Matrigel invasion assay: bright field microscopy images (100× magnification) of stained cells transmigrated −/+25 ng/ml TNFα are shown. **E**) Areas covered by control and TNFα stimulated TaC12 cells were quantified and are expressed as average spreading area per cell (n = 50 cells per group).
(TIF)

Figure S2 **Endogenous TNFα is not required for directional migration.** **A**) qRT-PCR analysis of TNFα mRNA expression of TaC12 cells 48 h after transfection with siTNFα_1 or siTNFα_2. Means −/+ SD are shown. **B**) Box plots of FMI (ratios of distance/path length) of siControl and siTNFα cells (n = 90 cells per group). **C**) Histogram shows frequencies of degrees of angular turns per step of siControl and siTNFα expressed in radians (n = 90 cells per group). **D**) Cells with single lameillipodia were quantified in Thei cells seeded on fibronectin 24 h after transfection with either siControl or siTNFα_1 or siTNFα_2.
(TIF)

Figure S3 **Presence of parasite affects MAP4K4 expression and kinase activity.** Quantification of Ib analyses of untreated and 48 h BW720c-treated Thei (**A**) or TaH12810 (**B**) cells with anti-MAP4K4, anti-ERM, anti-Hck and anti-tubulin antibodies. Quantifications of mean protein expression −/+ SD relative to tubulin are shown. 3 independent experiments. **C**) *In vitro* kinase assay using Myelin basic protein (MBP) as substrate and comparing MAP4K4 kinase activity immunoprecipitated either from infected or cured cells. Upper: Ib of immunoprecipitated MAP4K4, lower: autorad shows MBP phosphorylation. As comparison, MAP4K4-wt or MAP4K4-k/d were expressed in HEK293T cells and activities in the relative immunoprecipitates were compared in MB kinase assay.
(TIF)

Figure S4 **MAP4K4 is not required for directional migration.** **A**) Box plots of FMI (ratios of distance/path length) of siControl and siMAP4K4 cells (n = 60 cells per group). **B**) Histogram shows frequencies of degrees of angular turns per step expressed in radians of si-control and si-MAP4K4 (n = 60 cells per group). **C**) siCcontrol or siMAP4K4 TaC12 cells were embedded in matrigel and then stimulated or not with 5 ng/ml TNFα. Maximum intensity projections of 50–60 images over a z-range of a 150 µm are shown. **D**) Percentage of cells with protrusions shown in C was quantified from three randomly chosen fields.
(TIF)

Figure S5 **A) Treatment with Etoposide or nutlin promotes p53 nuclear accumulation in infected cells.** Confocal IFA analysis of p53 localization in TaC12 cells after 12 h of Etoposide (42 µM) or Nutlin (5 µM) treatment. Parasite (TaSP) is red, p53 green and host parasite and nuclear DNA is labeled with hoechst (blue). **B) Proteasome inhibition only partially rescues MAP4K4 abundance after BW720c treatment.** Upper: Ib analysis of MAP4K4, Hck and tubulin abundance in lysates of control and BW720c-treated cells kept for the indicated times in the presence of the proteasome inhibitor MG132. Lower: quantification of protein abundance relative to tubulin.
(TIF)

Figure S6 **pERM proteins in spike-like membrane protrusions in MAP4K4 depleted cells.** IFA of siControl or siMAP4K4 transfected cells. Actin is red, pERM green and nuclear DNA blue.
(TIF)

Figure S7 **Schema summarizing TNFα-induced and MAP4K4-dependent pathways contributing to motility and invasiveness of** ***Theileria annulata*** **transformed macrophages.**
(TIF)

Movie S1 **TaC12 cells were nucleofected with EGFP-NIKwt.** Cell behavior was monitored by DIC live-cell imaging 30 h after transfection. 1 h, 1 min intervals, acceleration: 900×.
(MOV)

Movie S2 **TaC12 cells were nucleofected with EGFP-NIKwt.** Cell behavior was monitored by fluorescence live-cell imaging 30 h after transfection. 1 h, 1 min intervals, acceleration: 900×.
(MOV)

Movie S3 **TaC12 cells were nucleofected with EGFP-NIKD152N.** Cell behavior was monitored by DIC live-cell imaging 30 h after transfection. 1 h, 1 min intervals, acceleration: 900×.
(MOV)

Movie S4 **TaC12 cells were nucleofected with EGFP-NIKD152N.** Cell behavior was monitored by fluorescence live-cell imaging 30 h after transfection. 1 h, 1 min intervals, acceleration: 900×.
(MOV)

Table S1 **TargetScan analysis of predicted miRNA binding sites in the** ***Bos taurus*** **and** ***Homo sapiens*** **MAP4K4 3′UTRs.**
(DOCX)
