# Supplementary figures and images for: Intracellular Theileria annulata Promote Invasive Cell Motility through Kinase Regulation of the Host Actin Cytoskeleton
Source: PLoS Pathog. 2014 Mar 13;10(3):e1004003. doi: 10.1371/journal.ppat.1004003 (PMC3953445; doi:10.1371/journal.ppat.1004003)

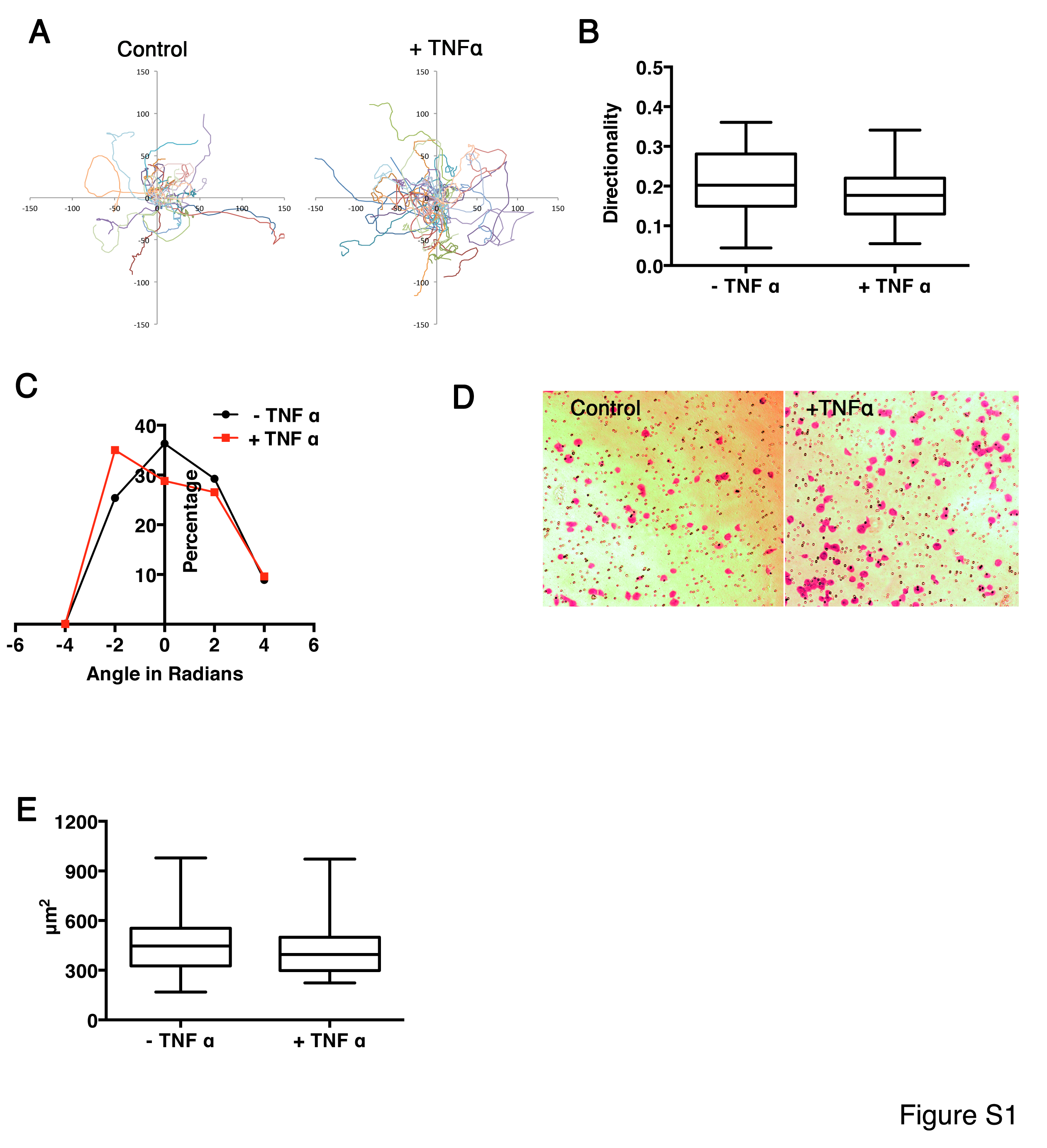

Supplement: Figure S1 — Exogenous TNFα does not affect cell spreading or directionality of migration. A) Tracks of single TaC12 cells in the absence (control) or presence of exogenous TNFα (25 ng/ml). B) Box plots of FMI (ratios of distance/path length) of control and TNFα-stimulated cells (n = 90 cells per group). C) Histogram shows frequencies of degrees of angular turns per step of control and TNFα-stimulated cells expressed in radians (n = 90 cells per group). D) Matrigel invasion assay: bright field microscopy images (100× magnification) of stained cells transmigrated −/+25 ng/ml TNFα are shown. E) Areas covered by control and TNFα stimulated TaC12 cells were quantified and are expressed as average spreading area per cell (n = 50 cells per group). (TIF) [file ppat.1004003.s001.tif]

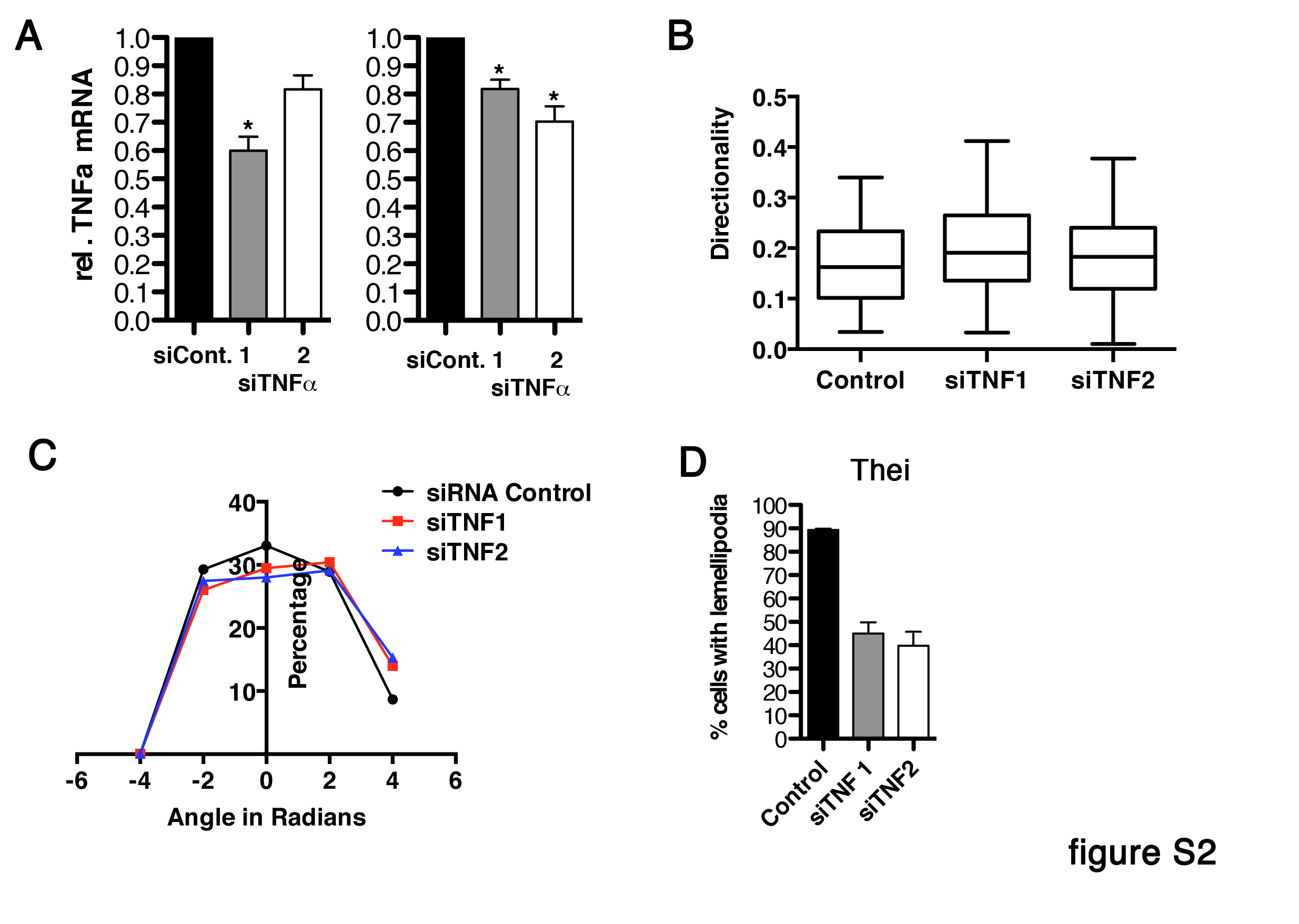

Supplement: Figure S2 — Endogenous TNFα is not required for directional migration. A) qRT-PCR analysis of TNFα mRNA expression of TaC12 cells 48 h after transfection with siTNFα_1 or siTNFα_2. Means −/+ SD are shown. B) Box plots of FMI (ratios of distance/path length) of siControl and siTNFα cells (n = 90 cells per group). C) Histogram shows frequencies of degrees of angular turns per step of siControl and siTNFα expressed in radians (n = 90 cells per group). D) Cells with single lameillipodia were quantified in Thei cells seeded on fibronectin 24 h after transfection with either siControl or siTNFα_1 or siTNFα_2. (TIF) [file ppat.1004003.s002.tif]

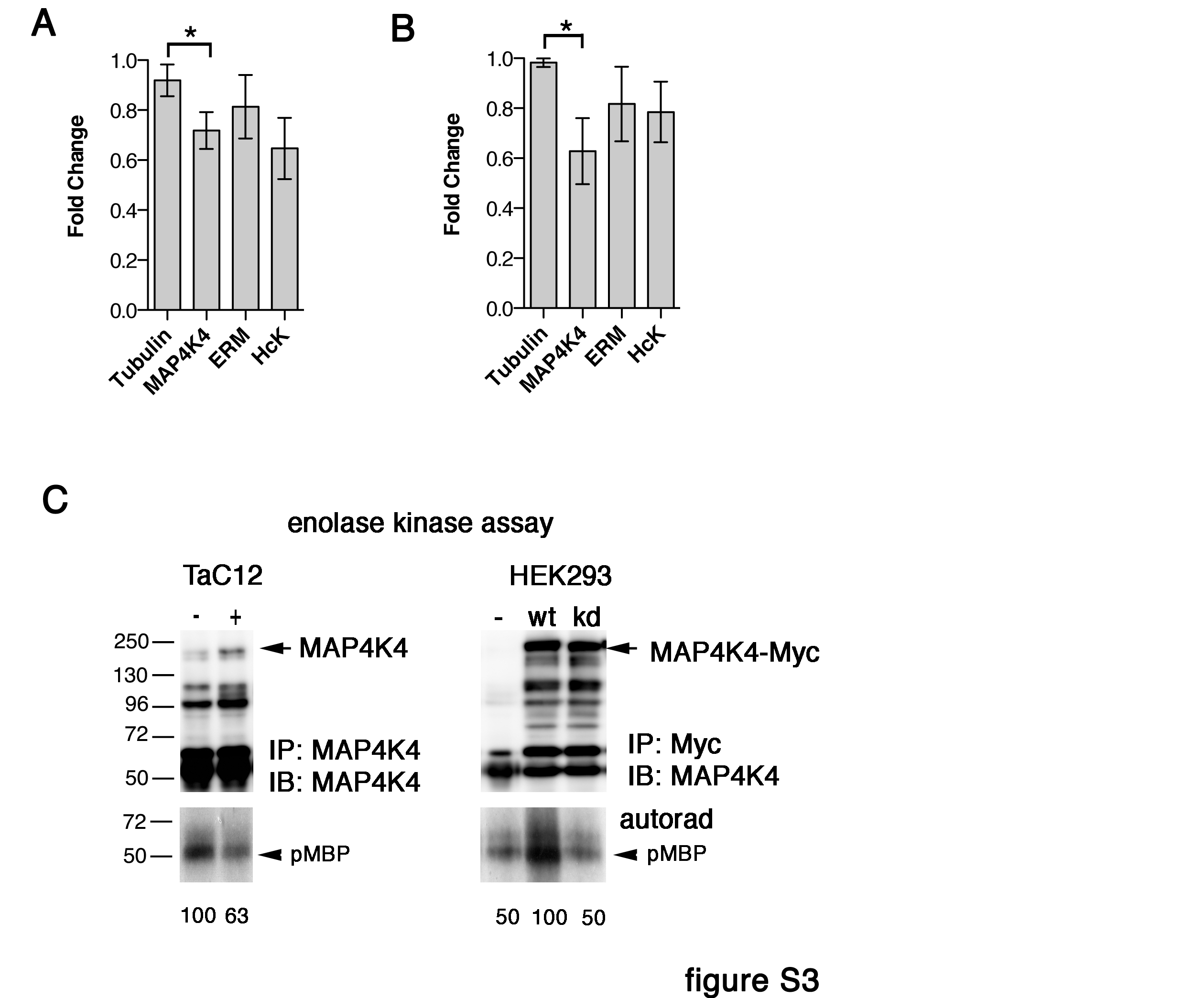

Supplement: Figure S3 — Presence of parasite affects MAP4K4 expression and kinase activity. Quantification of Ib analyses of untreated and 48 h BW720c-treated Thei (A) or TaH12810 (B) cells with anti-MAP4K4, anti-ERM, anti-Hck and anti-tubulin antibodies. Quantifications of mean protein expression −/+ SD relative to tubulin are shown. 3 independent experiments. C) In vitro kinase assay using Myelin basic protein (MBP) as substrate and comparing MAP4K4 kinase activity immunoprecipitated either from infected or cured cells. Upper: Ib of immunoprecipitated MAP4K4, lower: autorad shows MBP phosphorylation. As comparison, MAP4K4-wt or MAP4K4-k/d were expressed in HEK293T cells and activities in the relative immunoprecipitates were compared in MB kinase assay. (TIF) [file ppat.1004003.s003.tif]

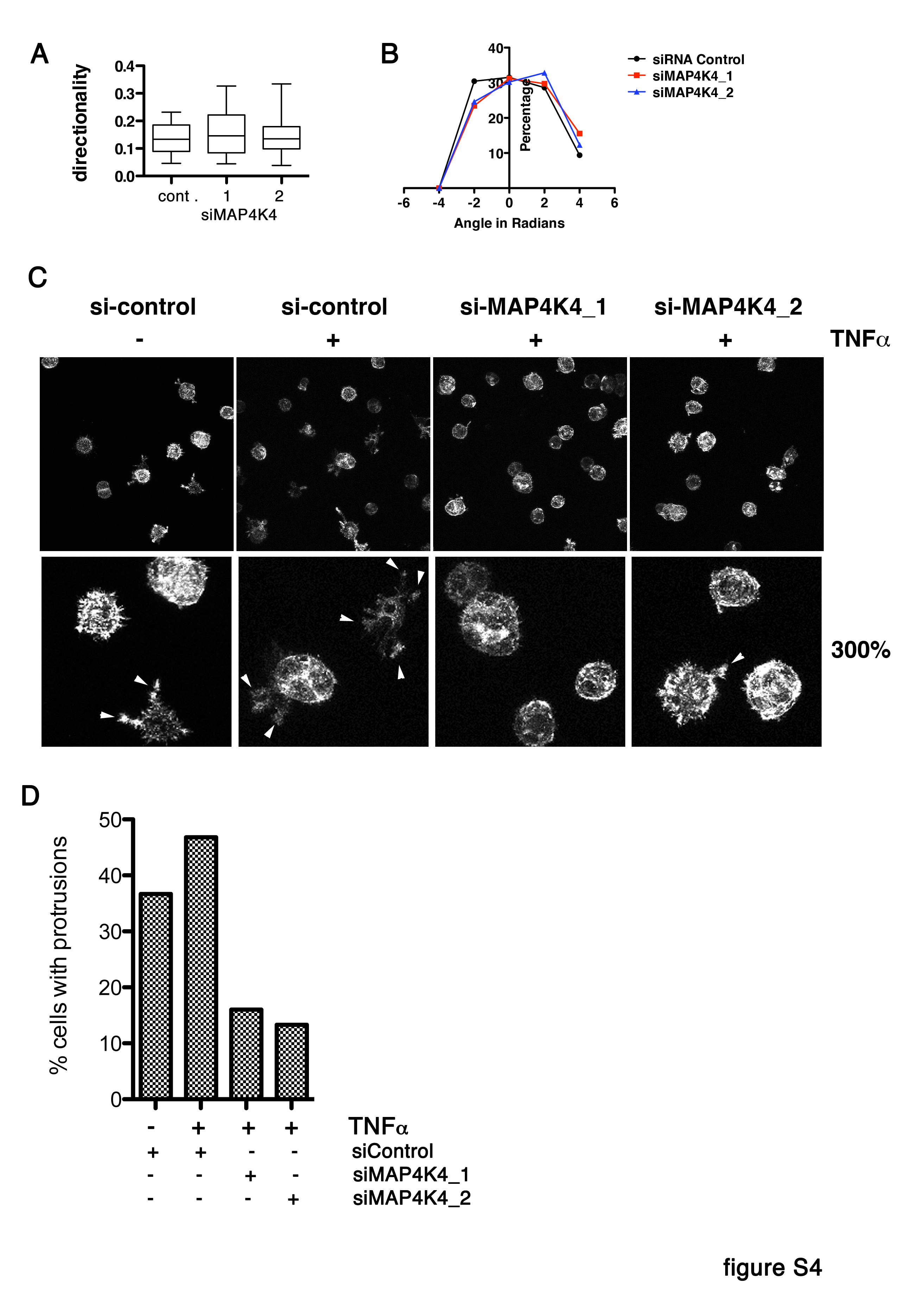

Supplement: Figure S4 — MAP4K4 is not required for directional migration. A) Box plots of FMI (ratios of distance/path length) of siControl and siMAP4K4 cells (n = 60 cells per group). B) Histogram shows frequencies of degrees of angular turns per step expressed in radians of si-control and si-MAP4K4 (n = 60 cells per group). C) siCcontrol or siMAP4K4 TaC12 cells were embedded in matrigel and then stimulated or not with 5 ng/ml TNFα. Maximum intensity projections of 50–60 images over a z-range of a 150 µm are shown. D) Percentage of cells with protrusions shown in C was quantified from three randomly chosen fields. (TIF) [file ppat.1004003.s004.tif]

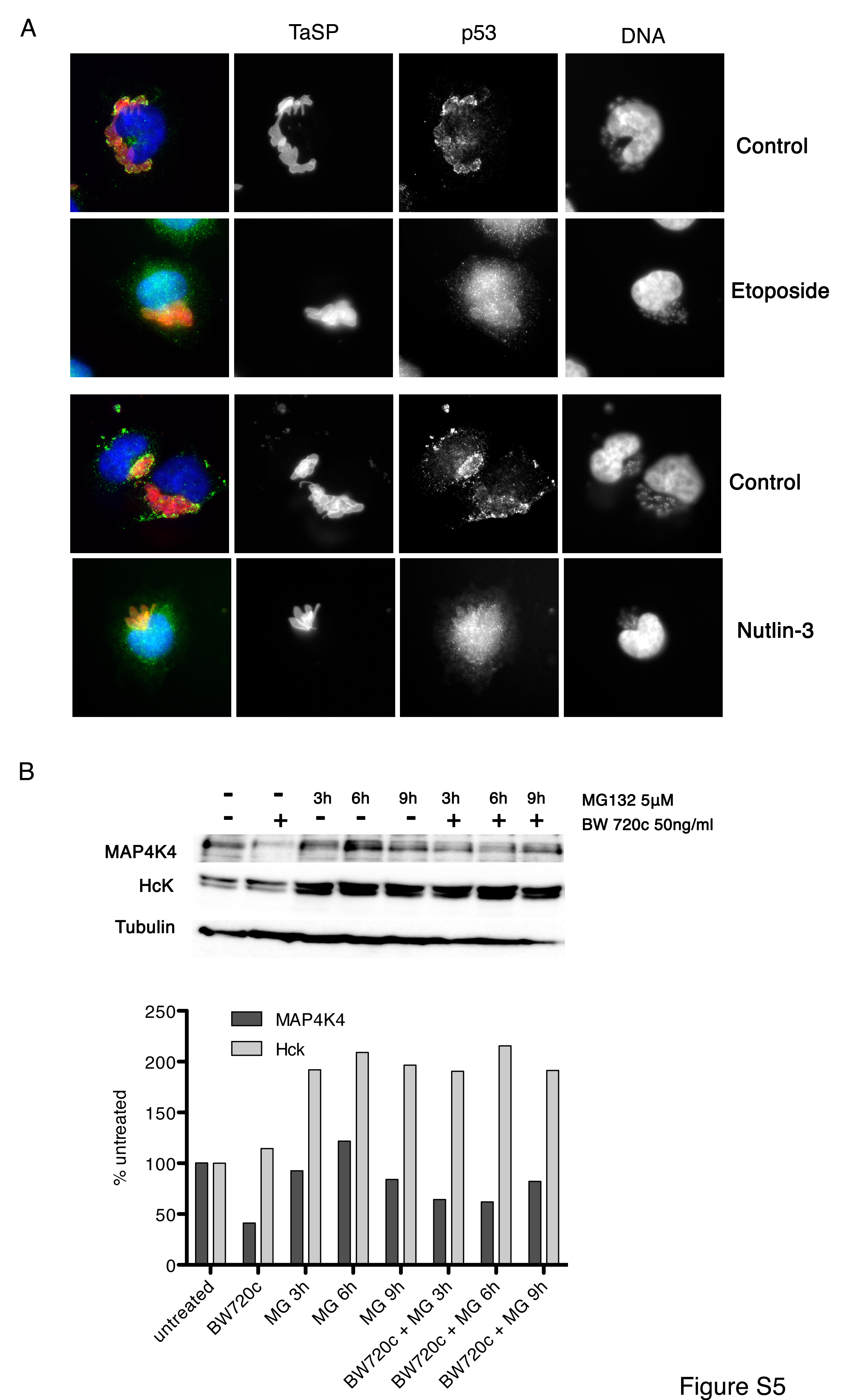

Supplement: Figure S5 — A) Treatment with Etoposide or nutlin promotes p53 nuclear accumulation in infected cells. Confocal IFA analysis of p53 localization in TaC12 cells after 12 h of Etoposide (42 µM) or Nutlin (5 µM) treatment. Parasite (TaSP) is red, p53 green and host parasite and nuclear DNA is labeled with hoechst (blue). B) Proteasome inhibition only partially rescues MAP4K4 abundance after BW720c treatment. Upper: Ib analysis of MAP4K4, Hck and tubulin abundance in lysates of control and BW720c-treated cells kept for the indicated times in the presence of the proteasome inhibitor MG132. Lower: quantification of protein abundance relative to tubulin. (TIF) [file ppat.1004003.s005.tif]

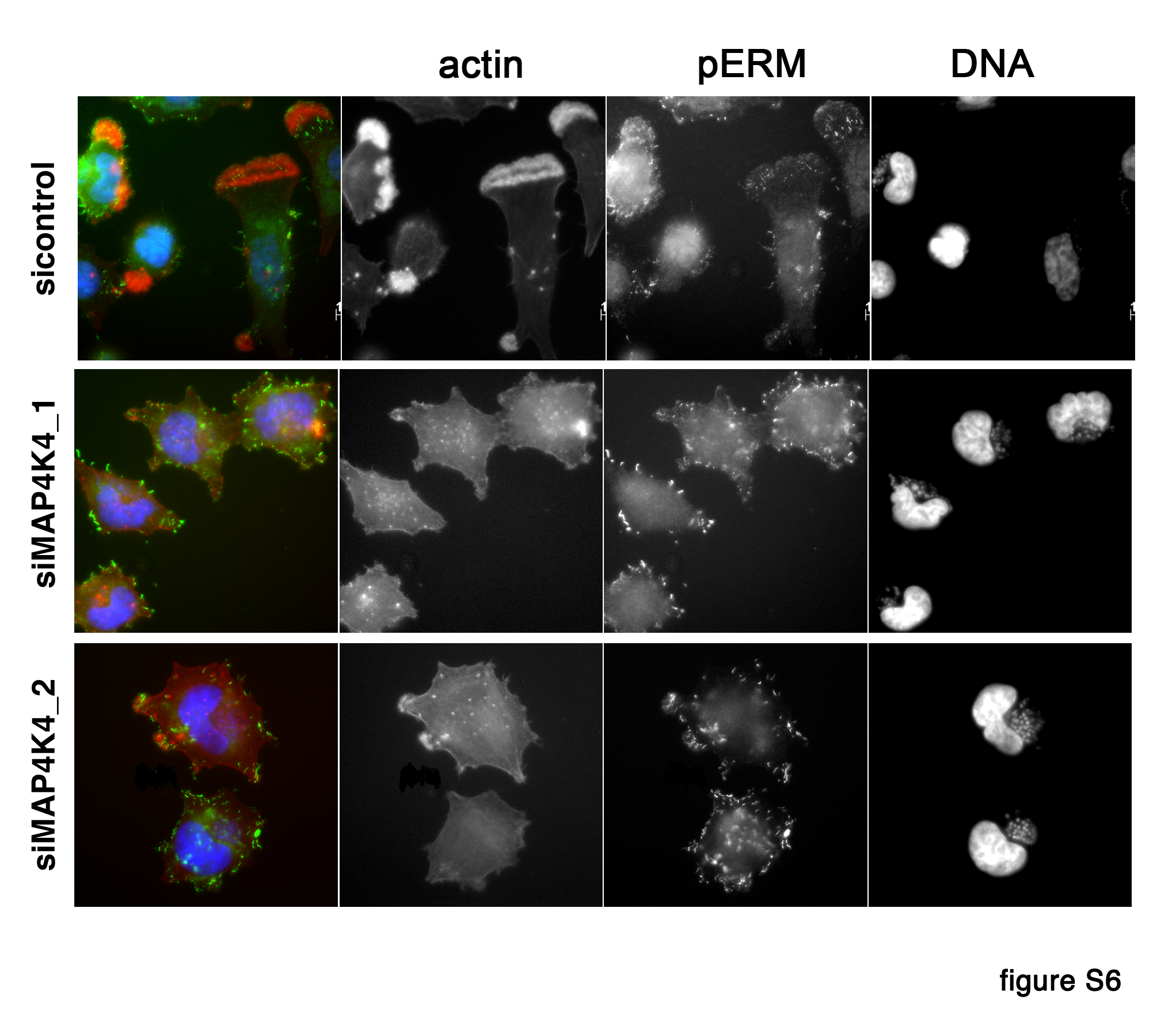

Supplement: Figure S6 — pERM proteins in spike-like membrane protrusions in MAP4K4 depleted cells. IFA of siControl or siMAP4K4 transfected cells. Actin is red, pERM green and nuclear DNA blue. (TIF) [file ppat.1004003.s006.tif]

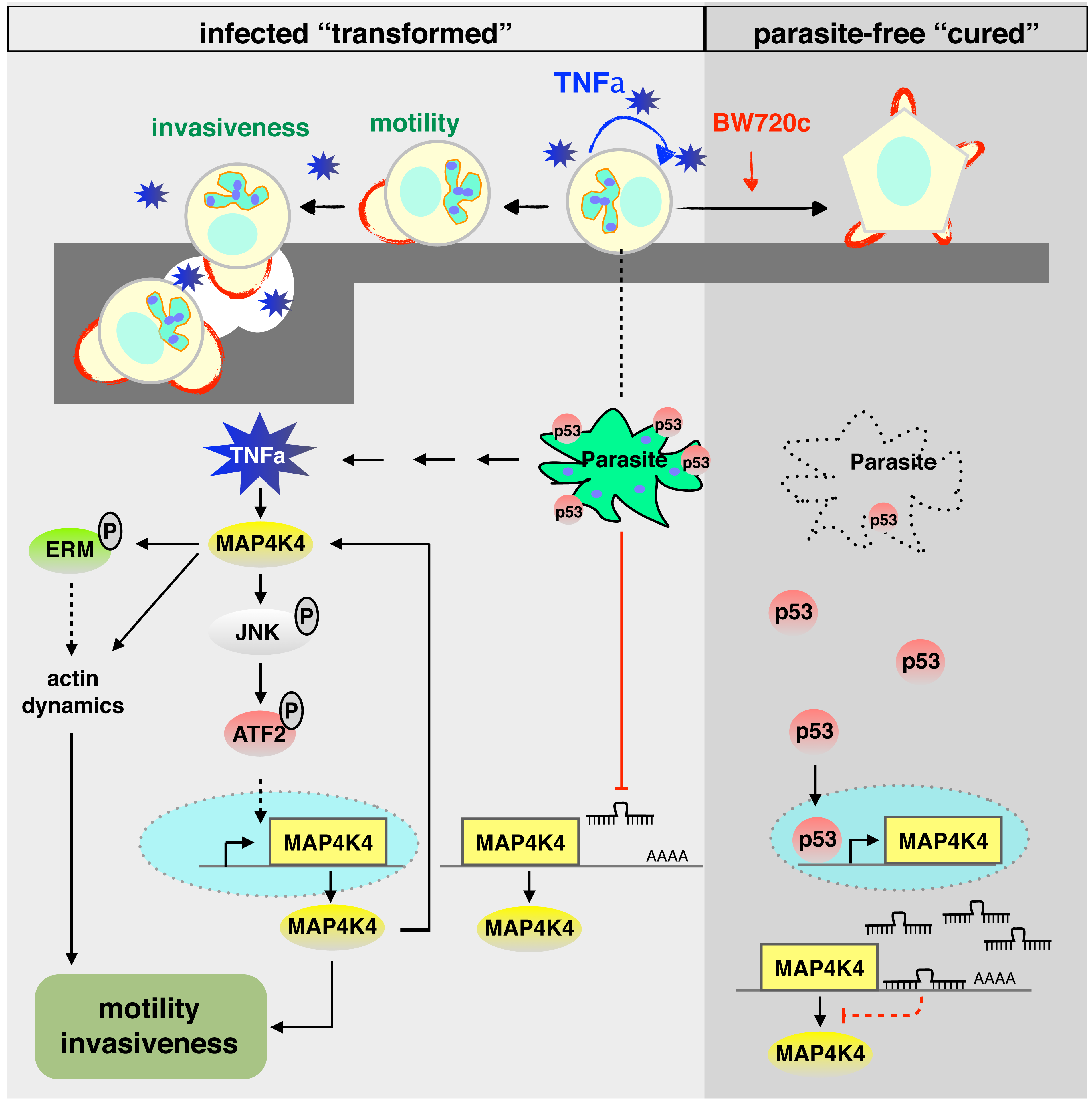

Supplement: Figure S7 — Schema summarizing TNFα-induced and MAP4K4-dependent pathways contributing to motility and invasiveness of Theileria annulata transformed macrophages. (TIF) [file ppat.1004003.s007.tif]
