# Supplementary material for: Intracellular Theileria annulata Promote Invasive Cell Motility through Kinase Regulation of the Host Actin Cytoskeleton
Source: PLoS Pathog. 2014 Mar 13;10(3):e1004003. doi: 10.1371/journal.ppat.1004003 (PMC3953445; doi:10.1371/journal.ppat.1004003)
Supplement: Table S1 — TargetScan analysis of predicted miRNA binding sites in the Bos taurus and Homo sapiens MAP4K4 3′UTRs. (DOCX) [file ppat.1004003.s012.docx]

**Supporting Table S1:**

|  | **miRNA** | **Conserved hs versus bt** |
| --- | --- | --- |
| **miRNA binding sites shared by human and bovine MAP4K4 3'UTR** | miR-29abcd | yes |
|  | miR-200bc | yes |
|  | miR-130ac | yes |
|  | miR-301ab | yes |
|  | miR-301b | yes |
|  | miR-301b-3p | yes |
|  | miR-454 | yes |
|  | miR-721 | yes |
|  | miR-4295 | yes |
|  | miR-3666 | yes |
|  | miR-30abcdef | yes |
|  | miR-30abe-5p | yes |
|  | miR-96 | yes |
|  | miR-507 | yes |
|  | miR-1271 | yes |
|  | miR-548 | yes |
|  | let-7 | yes |
|  | let-98 | yes |
|  | let-4458 | yes |
|  | let-4500 | yes |
|  | miR-29abcd | yes |
|  | miR-145 | yes |
|  | miR-33ab | yes |
|  | miR-33-5p | yes |
|  | miR-155 | yes |
|  | miR-194 | yes |
|  | mir-181abcd | yes |
|  | miR-4262 | yes |
|  | miR-10abc/10a-5p | yes |
|  | miR-10a-5p | yes |
|  | miR-155 | yes |
|  | miR-429 | yes |
|  | miR-548 | yes |
| **only on bt MAP4K4 3'UTR:** | miR-132 | no |
|  | miR-212 | no |
|  | miR-212-3p | no |
| **Only on hs MAP4K4 3’UTR** | miR-449abc | yes |
|  | miR-449c-5p | yes |
|  | miR-34ac/34bc | yes |
|  | miR-10abc//10a-5p | yes |
|  | miR-202-3p | yes |
|  | miR-495 | yes |
|  | miR-1192 | yes |
|  | miR-590-3p | yes |
|  | miR-141/200a | no |
|  | miR-340-5p | no |
|  | miR-23abc | no |
|  | miR-3p | no |
